# Personalised exercise therapy and self-management support for people with multimorbidity: Development of the MOBILIZE intervention

**DOI:** 10.1186/s40814-022-01204-y

**Published:** 2022-12-02

**Authors:** Alessio Bricca, Madalina Jäger, Mette Dideriksen, Hanne Rasmussen, Mette Nyberg, Julie Rønne Pedersen, Graziella Zangger, Karen Hjerrild Andreasson, Søren T. Skou

**Affiliations:** 1 Research Unit for Musculoskeletal Function and Physiotherapy, Department of Sports Science and Clinical Biomechanics, University of Southern Denmark, 5230 Odense M, Denmark; 2 The Research Unit PROgrez, Department of Physiotherapy and Occupational Therapy, Næstved-Slagelse-Ringsted Hospitals, Slagelse, Denmark; 3 Danish centre for motivation and behaviour science, Department of Sports Science and Clinical Biomechanics, University of Southern Denmark, Odense M, Denmark

**Keywords:** Multimorbidity, Exercise, Self-management, Intervention Development, Quality of Life, Physical Function, Feasibility

## Abstract

**Background:**

To our knowledge, there is no intervention which includes personalised exercise therapy and self-management support for people with multimorbidity, although these interventions may be as effective as for people with single chronic conditions. Therefore, we developed a novel intervention, including personalised exercise therapy and self-management support for people with multimorbidity.

**Methods:**

We followed the Medical Research Council framework and conducted one scoping review, five systematic reviews, two registry-based studies, one qualitative interview study and a mixed-methods feasibility study. Following an iterative approach, together with feedback from people with multimorbidity and relevant stakeholders, we developed the MOBILIZE intervention.

**Results:**

The intervention included 24 (60 minutes) sessions of personalised exercise therapy and 24 (30 minutes) sessions of self-management support twice a week for 12 weeks, delivered in small groups by specifically trained physiotherapists. The intervention targets physiological, psychosocial, behavioural, and contextual factors to improve health-related quality of life and physical function in people living with multimorbidity.

**Conclusions:**

We developed a personalised exercise therapy and self-management support programme for people with multimorbidity. The intervention will be tested for its safety and effectiveness in a randomised controlled trial.

**Supplementary Information:**

The online version contains supplementary material available at 10.1186/s40814-022-01204-y.

## Key findings

- We developed a novel intervention for people with multimorbidity through an iterative and comprehensive process following the Medical Research Council framework. This included several reviews, cohort studies and interviews with people with multimorbidity and several stakeholders.
- The detailed reporting of the development of the intervention can serve as a model for future development papers to increase transparency and reduce research waste.
- We focused on people with specific combinations of long-term chronic conditions (i.e., multimorbidity), therefore, this intervention is tailored for this population.
- The intervention has been developed to be delivered in person, therefore, delivering this intervention using other delivery modalities (e.g., digitally) may require amendments.

## Background

Living with two or more chronic conditions (i.e., multimorbidity) is common among people of all ages [1, 2]. Worldwide, approximately one-third of the population lives with one or more chronic conditions [3]. People living with multimorbidity have poorer physical and psychosocial health, a higher risk of hospitalisation and of dying prematurely [4–7]. The complexity of multimorbidity challenges the single-disease approach generally used by healthcare sectors, where patients use several healthcare services to manage each of their long-term conditions separately [8, 9], which is inefficient and burdensome for the patient [10]. Furthermore, available evidence for effective interventions of multimorbidity is limited [11]. A possible solution to deal with the complexity of managing multimorbidity is to focus on specific combinations of conditions, linked by common risk factors [12–14]. This approach may also help improve care and treatment effects for this population [11].

Osteoarthritis (of the knee or hip), type 2 diabetes, depression, heart disease (heart failure or ischemic heart disease), hypertension, and chronic obstructive pulmonary disease [15–22], are among the leading causes of global disability, affect hundreds of millions of people around the world [23], and often coexist linked via systemic inflammation and physical inactivity [24, 25]. Treatment guidelines, while encouraging clinicians to recommend a healthy lifestyle (including exercise therapy and self-management) for people with multimorbidity, focus on pharmacology [26]. However, to our knowledge, there is no intervention which includes personalised exercise therapy and self-management support for people with multimorbidity, although these interventions may be as effective as for people with single chronic conditions [27–29].

Therefore, the aim of this paper is to describe the development of a novel intervention (MOBILIZE) which includes personalised exercise therapy and self-management for people with multimorbidity.

## Methods

The development of the MOBILIZE intervention is reported following the GUIDance for the rEporting of intervention Development (GUIDED) recommendations (S1 Table) [30]. We followed the newest version of the Medical Research Council Framework [31] to develop the intervention supplemented with crucial elements from the Bleijenberg et al. [32] and O'Cathain et al. guidance [33]. This was done to improve the likelihood of developing an intervention feasible to deliver and accepted by patients living with multimorbidity to ultimately enhance the fit with clinical practice [31–33]. Overall, the development of the intervention can be summarised in four phases: (I) “evidence synthesis and registry-based studies” where we summarised the available evidence regarding the effect of exercise therapy and self-management for people with multimorbidity and identified predictors of health outcomes in such interventions, (II) “qualitative analyses” where we explored the perspectives of patients living with multimorbidity, health care professionals, relatives, and patient advocates in relation to self-management with a particular focus on exercise behaviour, (III) theories and mechanism of action for developing the intervention, where we hypothesised the potential mechanisms of action and created a logic model, and finally (IV) mixed-methods feasibility testing of the developed intervention. The full results of the feasibility study are reported elsewhere [34].

### Patient and Public Involvement

The development of the MOBILIZE intervention included feedback from several stakeholders, that is, people living with multimorbidity and their carers, patient advocates, physiotherapists and occupational therapists who are routinely working with patients with chronic conditions, a dietician, medical doctors specialised in the chronic conditions of interest, researchers with different backgrounds (physiotherapists, an exercise physiologist, and a health psychologist). All the stakeholders were informed about the different stages of the development and asked to provide feedback when deemed relevant. To determine how the body of evidence generated from the four phases of intervention development could be integrated into the intervention, the research team (AB, MJ, and STS) presented an initial program (based on the evidence gathered in phases (I) and (II)), to the physiotherapists (MD, HR, MN, JRP, GZ, and KHA), the patient advocates and carers, and medical doctors. The structure of the programme was discussed, including the exercises proposed, progression/regression levels, and self-management themes. This approach was also used throughout the development of the intervention, including during the feasibility study, and helped inform the final version of the intervention that is being tested in a randomised controlled trial (RCT) (https://clinicaltrials.gov/ct2/show/NCT04645732). This approach strengthens the internal and external validity, minimises research waste, and adds value to health care research [31–33]. The following sections describe the development process of the intervention and how the intervention was adapted after qualitative and quantitative data on the feasibility study were gathered.

## Results

**Fig. 1:**
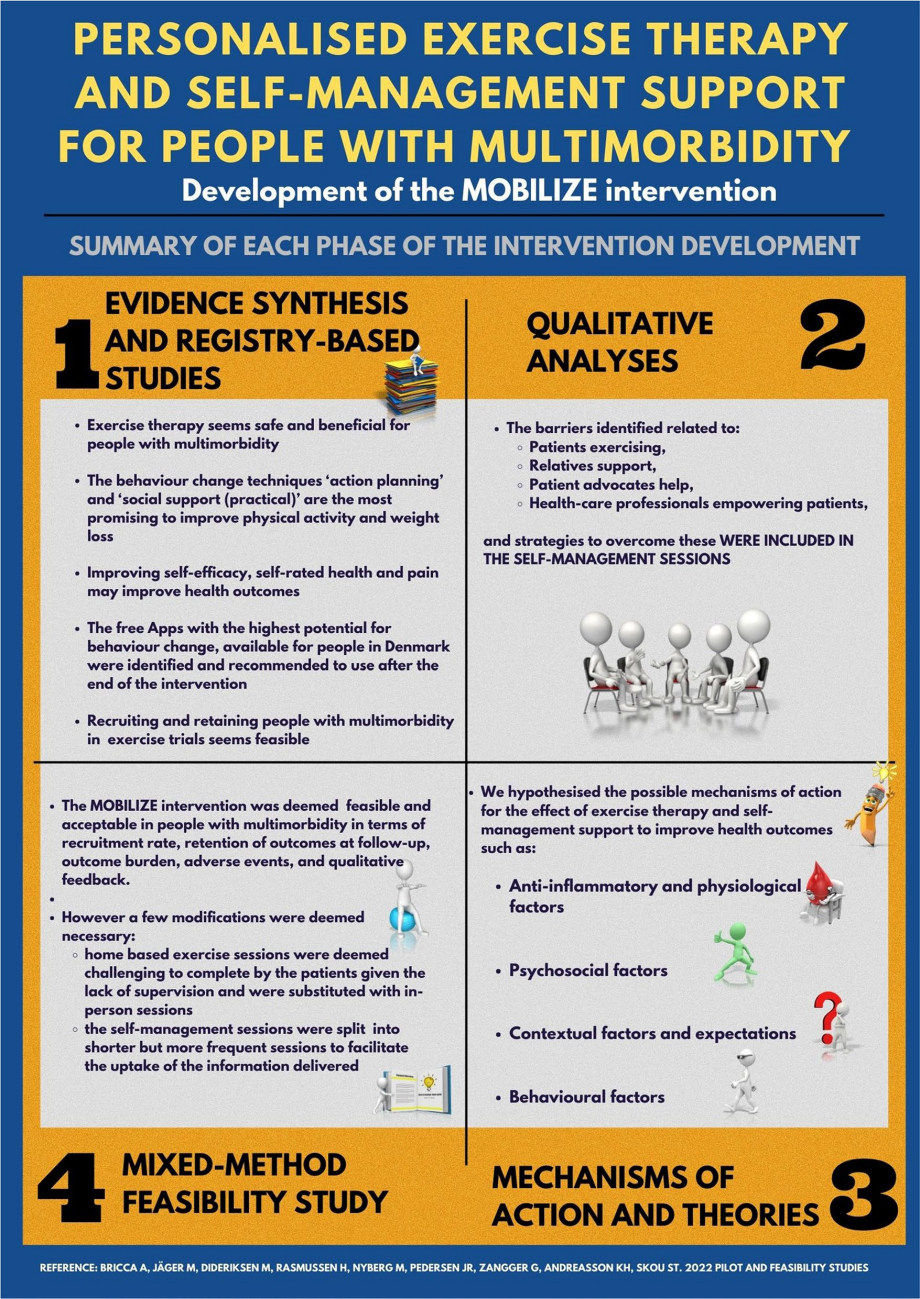
Infographic of the development process

Overall, we performed one scoping review [35], five systematic reviews [27, 36–40], two registry-based studies [41, 42], one qualitative interview study [43], and a mixed-methods feasibility study [34], to identify knowledge gaps and develop an exercise therapy and self-management intervention for people with multimorbidity (Table 1) and Fig. 1.

**Table 1:** Studies conducted in the MOBILIZE project to identify knowledge gaps in the design and conduct of exercise therapy and self-management trial in people with multimorbidity

Study and design	Aim of the study	Main findings	How this knowledge guided the development of the intervention
Bricca et al. 2019, 2020, 2021 (Systematic review protocol, systematic review, and infographic) [27, 36, 37]	To investigate the benefits and harms of exercise therapy for people with multimorbidity.	Exercise therapy appears to be safe and to have a beneficial effect on physical and psychosocial health in people with multimorbidity. Although the evidence supporting this was of low quality, it highlights the potential of exercise therapy in the management and care of this population.	Exercise therapy was deemed as a potentially beneficial intervention for people with multimorbidity supporting its use as part of an intervention to improve health in people with multimorbidity. Given that no superior type of exercise therapy type was found to improve health, we discussed with stakeholders and patient partners the optimal type, dose, and intensity of the programme, considering patient preferences and the fact that it was easy to deliver and implement in clinical practice. The intervention details are reported in the TIDieR checklist incorporating the CERT items (Table 4), and Toigo & Boutellier checklist (S2 Table).
Harris et al. 2020 (systematic review) [40]	To quantify recruitment, retention and differential retention rates and associated trial, participant, and intervention characteristics in randomised controlled trials (RCTs) evaluating the effect of exercise therapy in people with multimorbidity.	Three in four eligible people with multimorbidity were randomised to RCTs using exercise therapy, of which nine out of 10 provided end of treatment outcomes with no difference seen between the intervention and comparison groups. However, the results must be interpreted with caution due to large differences between the included studies.	When planning the recruitment and retention phase, we used this knowledge to identify how many people were needed to be screened to reach the target number of people included in the feasibility study.
Pihl et al. 2021 (registry based) [42]	To investigate if comorbidities are associated with change in health outcomes following an 8-week exercise and education programme in knee and hip osteoarthritis.	Health outcomes improved regardless of coexisting comorbidities. This means that comorbidities are not associated with worse nor better health outcomes following an 8-week exercise and education programme in individuals with osteoarthritis, suggesting exercise as a viable treatment option for individuals with osteoarthritis, irrespective of comorbidities.	This knowledge supported the assumption that exercise therapy may be beneficial across people with several combinations of conditions, and no specific adaptation needs to be made for the subgroup of patients with OA and another condition.
Pihl et al. 2021 (registry based) [41]	To identify prognostic factors for health outcomes following an 8-week supervised exercise therapy and education programme for individuals with knee and hip osteoarthritis alone or with concomitant hypertension, heart or respiratory disease, diabetes, or depression.	Age, self-efficacy, self-rated health, and pain intensity may be prognostic of change in health outcomes following an 8-week exercise therapy and patient education programme for individuals with OA, irrespective of comorbidities.	We focused our self-management support programme on providing tools to improve self-efficacy, self-rated health and pain. The themes for the 24 self-management sessions are reported in Table 2.
Bricca et al. 2022 (systematic review) [39]	To assess the quality of health Apps and their potential for behaviour change.	Apps for people with a chronic condition or multimorbidity appear to be of acceptable quality but have low to moderate potential for promoting behaviour change.	We selected the highest (free) quality health Apps, and those with the highest potential for behaviour change, available for people in Denmark and provide practical support for downloading or using such Apps during one of the self-management sessions.
Jäger et al. 2022 (scoping review) (under review) [35]	To map the literature on patient-centred interventions for people living with multimorbidity that supports self-management.	The results pointed to an extensive use of cognitive behavioural therapy as a basis for interventions, as well as behaviour change theories and chronic disease management frameworks. The most coded Behaviour Change Techniques (BCTs) stemmed from the categories Social Support, Feedback and Monitoring, and Goals and Planning.	We considered which of the several BCTs used in RCTs were the most effective to promote behaviour change and which ones could be implemented in the clinical setting in which the intervention would be tested.
Bricca et al. 2022 (Systematic review) [38]	To investigate the effect of behavioural interventions in people with multimorbidity and to identify Behaviour Change Techniques (BCTs) associated with better outcomes.	Behavioural interventions targeting lifestyle behaviours may improve health-related quality of life and physical function, and reduce depression symptoms, whereas little to no effect was achieved on physical activity and weight loss in people with multimorbidity. However, the evidence for physical activity and weight loss was of low quality and the end-treatment benefits diminished over time. Yet, studies using the BCTs ‘action planning’ and ‘social support (practical)’ reported greater physical activity and weight loss.	These findings guided the developed of the the self-management sessions related to the importance of physical activity and how to implement it in daily routines (Table 3).
Zangger et al. 2022 (systematic review) (under review)	To assess the effect of digital health solutions promoting physical activity among people with one or more chronic conditions	Overall, using a digital health solution shows a small improvement in physical activity, physical function, quality of life, and a small reduction in depressive symptoms, but no change in anxiety symptoms compared to usual care.	We found low quality of evidence for the effect of digital solutions in promoting physical activity and improving health in people with multimorbidity. This knowledge guided the development of the self-management sessions related to self-monitoring (Table 3).

### Evidence synthesis

The reviews performed used the following Population, Intervention, Comparator and Outcome characteristics:

#### Population

Multimorbidity was defined as people reporting two or more of the following conditions: osteoarthritis (of the knee or hip), type 2 diabetes, depression, heart disease (heart failure or ischemic heart disease), hypertension, and chronic obstructive pulmonary disease. These conditions are linked by a common risk factor (physical inactivity) and pathogenesis (systemic low-grade inflammation), resulting in a cascade of reactions resulting in a ‘vicious cycle’ of chronic diseases and poor outcomes [44, 45]. For two systematic reviews, we included studies reporting at least 80% of the patients with multimorbidity (as defined above) [27, 40]. This pragmatic approach was pre-specified [37], and adopted to capture all the studies which included people with multimorbidity, given the expected inconsistency of reporting of the conditions across trials.

#### Interventions

Interventions that included exercise therapy and self-management support, either alone or in combination. We did not apply restrictions to the mode of delivery of the interventions (e.g., digitally, face-to-face) and setting (e.g., municipality/community, hospital).

#### Comparators

Usual care, for instance, advice from their health care provider.

#### Outcomes

The outcomes of interest included physical (e.g. endurance), psychosocial (e.g. health-related quality of life) and behavioural health (e.g. physical activity). The rationale for including these outcomes follows the guidance of a consensus study [14], which identified outcomes for multimorbidity intervention studies, and the patient partners of MOBILIZE.

### Registry-based studies

Additionally, two registry-based studies were conducted to investigate the impact of comorbidities on health outcomes and to identify prognostic factors for health outcomes following an 8-week exercise and education programme in people with knee or hip osteoarthritis [41, 42]. How the results of these studies informed the development of the intervention are summarised in Table 1.

### Qualitative analyses

A qualitative approach is particularly useful in capturing how people are affected by a problem and their perspectives on it [33]. Therefore, the qualitative study we conducted aimed to explore the perspectives of people living with multimorbidity, health care professionals, relatives, and patient advocates concerning self-management with a particular focus on exercise behaviour [43]. We conducted 17 interviews (nine focus groups; eight key informants) with 48 informants from four groups (22 people living with multimorbidity, 17 health care professionals, five relatives, and five patient advocates). The interviews were carried out online, informed consent was provided by the participants; the interviews were audio recorded and then transcribed verbatim. For the qualitative and feasibility study [34], patients and relatives were recruited by healthcare professionals from one psychiatric hospital and four hospital departments in Region Zealand (one of five health care regions in Denmark) or via self-referral on the basis of a poster and flyers placed in the hospitals’ waiting rooms or through posts on the hospitals’ and patient organizations’ Facebook pages [43]. The focus groups and interviews were audio-recorded, transcribed verbatim and analysed in an inductive-deductive process using Framework Analysis [46] and the Capability Opportunity and Motivation-Behaviour (COM-B) profile [47]. ,We found that people with multimorbidity identified several barriers related to exercise behaviour (i.e., pain, fatigue, breathlessness, lack of motivation, financial issues, accessibility, transportation, and decreased social support). Relatives' perspectives illustrated an uncertainty concerning their role in supporting self-management while simultaneously showing that they often take over responsibilities, which may represent a burden on their own wellbeing. Therefore, strategies for overcoming barriers to exercise and the participation of relatives in the self-management sessions, as part of usual care, were included in the programme (Table 2). Furthermore, patient advocates emphasised a need for more resources, such as establishing new collaborations and initiatives for people with multimorbidity and the lack of a 'burning platform' for multimorbidity (i.e., lack of urgency and prioritization of multimorbidity in the society) . Hence, this was included in the self-management session by informing the patients about activities, events, and organisations they could join (Table 2). Finally, health care professionals recognised these challenges while sharing their own challenges of empowering people with multimorbidity to change their behaviour given the limited resources. Therefore, this implied we instructed the physiotherapists delivering the intervention to prioritise strategies (i.e., behaviour change techniques) shown to be associated with better health outcomes in people with multimorbidity (Table 1) [38]. Overall, this knowledge, together with the self-management support framework [48], served to identify the core element of the 24 themes of the self-management sessions as well as the specific content of the sessions (Table 2). The core elements of the self-management sessions were proposed by the author team to the patients and physiotherapists delivering the intervention, and this format was deemed acceptable and feasible by them.

**Table 2:** Self-management themes of the 24, 30 minute sessions of the MOBILIZE intervention. Since participants are enrolled on a rolling basis, they might receive the sessions in a different order

Theme	Short description of the session
Self-management	Self-management and taking responsibility for own health and wellbeing
Physical activity	The importance of physical activity and how to implement it in daily routines
Mindfulness	Introduction to mindfulness: a tool to help connect to the present moment
Your story	Personal journey of living with multiple chronic conditions and learning other patients’ stories
General physical symptoms	Successfully handling physical symptoms related to chronic conditions
Healthy eating habits	Healthy food choices and maintaining a healthy diet
Patient relatives (relatives or carers of the patients are invited to participate in the session)	The role of relatives in practical and emotional support and how to handle the complexity of patient-relative interactions
Reactions to chronic illness	Common stress reactions and the way they influence health
Breathing as a tool	Simple breathing techniques for stress relief
Physical exercise	The role and importance of strengthening and aerobic exercises
Mindfulness: body scan	Mindfulness session: Body scan for relaxation and body awareness
General mental symptoms	Handling the mental health aspects of living with chronic conditions
Coping strategies	Introduction to different coping strategies and how to use them
Goal setting (relatives or carers of the patients are invited to participate in the session)	Setting personal goals and overcoming obstacles
Breaking down barriers to physical activity	Identification of barriers to physical activity and exercise and how to overcome them
Pain mechanisms	Pain mechanisms and contributors to living a meaningful life with chronic pain
Communication	Communicating effectively with health care professionals
Exercise and chronic conditions	Safety when exercising with chronic conditions
Mindful eating	Eating habits awareness and practising mindful eating
Breathing and shortness of breath (breathlessness)	The mechanisms of breathing and the difference between shortness of breath and difficulty of breathing (dyspnoea)
Self-care	Self-care and active management of chronic conditions
Self-monitoring	Pros and cons of self-monitoring and how to get started
Mindfulness techniques	Mindful techniques that help when feeling overwhelmed
After MOBILIZE (relatives or carers of the patients are invited to participate in the session)	How to proceed after MOBILIZE: understand how to maintain improvements after the completion of the intervention

### Mechanism of action and theory to inform the development of the intervention

**Fig. 2:**
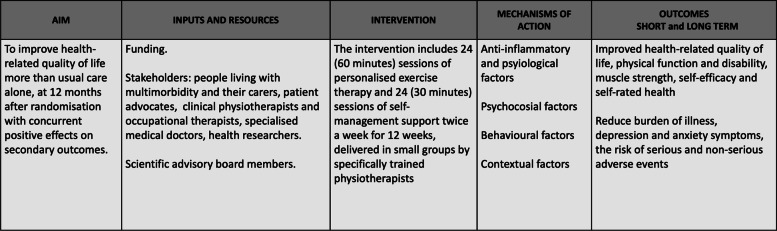
Logic model for the MOBILIZE intervention

In this section, we report the possible mechanisms of action of the MOBILIZE intervention, which are summarised in Fig. 2. Overall, we hypothesised that personalised exercise therapy and self-management support in addition to usual care (for instance, advice from their health care provider or any other treatment prescribed as standard care) will improve health-related quality of life more than usual care alone when measured at 12 months with concurrent positive effects on secondary outcomes. Furthermore, we hypothesised those improvements will occur immediately after the programme with concurrent positive effects on secondary outcomes and that the programme will be cost-effective at 12 months. We, therefore, aimed to develop an intervention that may potentially generate these effects Fig. 2. The knowledge gained from the four phases of intervention development guided us in identifying the core components of the exercise therapy and self-management intervention, as summarised in Table 1.

### Anti-inflammatory and physiological factors

The anti-inflammatory effects of exercise therapy at cellular, tissue and organ level [44], as well as its positive physiological effects such as increase in muscle strength, improved blood pressure regulation and insulin sensitivity [49], highlight its potential role in improving health by reducing systemic inflammation.

### Psychosocial factors

Providing education about long-term chronic conditions, providing psychological and social support to adjust to life with chronic conditions, encouraging adherence to multiple treatments, and supporting activities of daily living and physical functioning may improve psychosocial wellbeing [50, 51]. This can be achieved both through exercise therapy amd self-management sessions and as result of the psychological and social benefits associated with participation in group-based activities [51].

### Contextual factors and expectations

Contextual factors, including the setting of the intervention [52], facilitator, patient and treatment features, as well as the patient-facilitator relationship [53], can influence health outcomes positively or negatively. Therefore, much attention is given to ensuring such factors are addressed when delivering the intervention. The complete list of contextual factors and how they are considered in the MOBILIZE intervention can be found in the S3 Table. Furthermore, expectations are a large determinant of the placebo and nocebo effect [54]. A feasible strategy to increase patients’ realistic expectations is making sure the patients are aware of the health benefits of exercise therapy and self-management. We have therefore instructed the facilitators delivering the intervention to use strategies to set realistic expectations [54]. For instance, via adapting an authentic and empathic communication style when communicating and via educating patients on how to cope with possible adverse effects (e.g. muscle pain, fatigue, and shortness of breath), regularly assess and address patients’ anxieties, concerns, and treatment expectations, and provide adequate information regarding diseases, diagnoses, and treatments [54]. Taken together, the contextual factors and expectations offer an opportunity to integrate strategies which may stimulate placebo effects and prevent nocebo effects [53].

### Behavioural factors

Engaging in a healthier lifestyle is associated with up to 6.3 years longer life expectancy for men and 7.6 years for women highlighting the role of a healthy lifestyle as a contributing factor to health [55]. Therefore, we included various Behaviour Change Techniques (BCTs) [56] in the intervention (Table 3). BCTs are defined as observable, replicable, and irreducible components of an intervention designed to alter or redirect causal processes that regulate behaviour. The rationale for using specific BCTs is based on the evidence synthesis results focusing on people with multimorbidity (Table 1) and previous literature investigating this topic given the low-certainty of the evidence for the association between BCTs and people with multimorbidity. For example, BCTs such as ‘Action planning’, ‘Self-monitoring’ and ‘Goal setting’ [56], are strongly associated with improved health behaviours in people without chronic conditions [57], and in people with one condition [55, 58–60].

**Table 3:** Behaviour Change Techniques (BCTs) used in the MOBILIZE intervention and examples of their use

BCT number and name [56]	Example of how the BCT is used in the intervention and (where)
1.1 Goal setting (behaviour)	Setting a Specific, Measurable, Attainable, Relevant, and Timely (SMART) goals (self-management sessions)
1.2 Problem solving	Prompting the patients to identify barriers to exercise (self-management sessions)
1.4 Action planning	Prompting exercising/being physically active (self-management and exercise therapy sessions)
1.5 Review behaviour goal(s)	Reviewing the SMART goal (self-management sessions)
2.3 Self-monitoring of behaviour	Provide the patients with a diary to record exercise sessions and exertion (exercise therapy sessions)
2.4 Self-monitoring of outcome(s) of behaviour	Ask the patients to record the exercise sessions and exertion (exercise therapy sessions)
3.1 Social support (unspecified)	Providing information about patient organisations and community resources that offer support (self-management sessions)
3.3 Social support (emotional)	Arranging emotional support from relatives or carers by inviting them to attend three self-management sessions (self-management sessions)
4.1 Instructions on how to perform a behaviour	Instructions on how to perform mindfulness exercises correctly (self-management sessions) and instructions on how to perform the physical exercises correctly (exercise therapy sessions)
5.1 Information about health consequences	Information about the positive effects of exercise, healthy eating and self-care (self-management sessions)
5.6 Information about emotional consequences	Information about the positive effects of exercising and a healthy lifestyle behaviour on health (self-management sessions)
6.1 Demonstration of the behaviour	Mindful eating exercise (self-management sessions) and demonstration of the exercises (exercise therapy sessions)
8.7 Graded tasks	Exercise progression (exercise therapy sessions)
9.1 Credible sources	The intervention is delivered by health care professionals (self-management and exercise therapy sessions)
10.4 Social reward	The facilitator of the interventions congratulates the patients when performing the exercises correctly and when attending the sessions (self-management and exercise therapy sessions)
11.2 Reduce negative emotions	Using stress management exercises to reduce stress and anxiety (self-management sessions)
12.6 Body changes	Prompt strengthening exercises (self-management and exercise therapy sessions)
15.1 Verbal persuasion about capability	The facilitators are instructed to persuade the patients that they can perform the exercises asserting that they can and will succeed (exercise therapy sessions)

In line with the BCT taxonomy instructions, we have only included the ‘active ingredients of the intervention’. That is, the observable, replicable components aimed at changing a behaviour.

### Personalisation of the MOBILIZE intervention

Prescribing the right treatment at the right time to the right patient is key to eliciting benefits and minimising harms. The exercise therapy and self-management content of the intervention has been developed to be personalised. The personalisation of the programme is set during a one-to-one session prior to initiating the programme, lasting 60 minutes between each patient and a physiotherapist. The one-to-one session includes the following phases: (I) Presentation of the exercise therapy programme to the patient and selection of the appropriate starting exercise levels, (II) Presentation of the criteria for progression or regression of the exercises (e.g., the OMNI scale [61], and the Borg scale [62], and how sets and repetitions are progressed are presented so that the patient can indicate the degree of exertion of the exercises) and instructions on how to fill in the intervention diary to record the exertion of the exercises, (III) setting of a Specific, Measurable, Attainable, Relevant, and Timely (SMART) goal (e.g., being able to walk the stairs without discomfort).

### Preference and the content of the MOBILIZE intervention

Adherence to treatment is key to its effectiveness [63]. For people with multimorbidity, this is particularly challenging due to the high treatment burden they experience [64]. Having the option to receive treatments based on patient preferences may improve adherence to the treatment and ultimately improve health [65]. Therefore, the MOBILIZE intervention allows patients to choose the type of exercise therapy (aerobic training, strength training, or functional exercises) to perform (Table 4). This decision was supported by the fact that people with multimorbidity seem to benefit from exercise therapy interventions regardless of the type of exercise therapy [27], in line with the WHO guidelines for physical activity for general health [66].

**Table 4:** Details of the MOBILIZE intervention: Personalised exercise therapy and self-management support programme

Item	Description
**1. Brief name**	MOBILIZE Personalised exercise therapy and self-management support programme (RCT version)
**2. Why**	There are no interventions, including personalised exercise therapy and self-management support for people with multimorbidity, as highlighted by the evidence synthesys we performed, although such interventions are recommended by clinical guidelines for people with single long-term conditions.
**3. What-materials**	For the exercise therapy sessions, elastic bands, treadmills, stationary bikes, kettlebells, gym balls, step boards and mats were used. For the self-management programme, a PowerPoint presentation supported by a manual for clinicians describing the intervention and including links, references and materials for patients to support self-management were used. The programme only required exercise equipment available in most gyms of hospitals and municipalities, ensuring the feasibility of the future implementation of the intervention. The material will be made publicly available once finalised and evaluated in the randomised, controlled trial. The What-materials was informed by the evidence sysnthesys and qualitative interviews.
**4. What-procedures**	The programme consists of 24 supervised exercise therapy sessions and 24 self-management sessions during the 12 weeks. This was deemed feasible and acceptable by patients and physiotherapists delivering the intervention. Before commencing the 12-week exercise programme, the patients are invited for a one-to-one assessment lasting 60 minutes where the physiotherapist delivering the intervention introduces the participant to the exercise therapy programme and sets the starting level for the exercises and the targeted intensity. The 12-week programme is supervised to ensure that the patients learn a certain skill set to self-manage their conditions and to initiate the behaviour change when it comes to for example physical activity (i.e., supporting them in understanding how and why they should continue exercising and being physically active). The exercise therapy consists of a warm-up phase (e.g., on a stationary bike), followed by PART 1 (all together); Balance exercises and strengthening exercises for the lower and upper body, then PART 2 (participant's own choice); Aerobic training, strength training or functional exercises, and finally a cool-down phase. The self-management programme combines individual and group activities and includes educational materials and videos, experiential exercises, and group discussions to equip patients with knowledge about chronic conditions and enable them to develop better self-management skills, set personal goals, and make action plans, identify challenges, and overcome them. At the end of the 12 weeks, the patients receive a video message on how to stay physically active and have a healthy lifestyle. This complements the self-management session ‘After MOBILIZE’.
**5. Who-provided**	The exercise therapy and self-management programme are facilitated by physiotherapists, who received specific one-day training session and three online half-day sessions in study procedures, supported by the MOBILIZE team. This is in line with what is delivered in clinical practice in Denmark, for people with single chronic conditions.
**6. How**	Delivered face to face (in-person) in small groups (max five patients) and individually and using different materials and videos as described under section 4.
**7. Where**	In Region Zealand, Denmark: the Department of Physiotherapy and Occupational Therapy at Slagelse and Næstved Hospitals, a private practice physiotherapy clinic in Holbæk and at rehabilitation centres in the municipalities of Roskilde and Lolland.
**8. When and how much**	24 (30-minute) self-management sessions followed by 24 (60-minute) supervised exercise therapy sessions delivered across 12 weeks with two sessions per week. Dosage (e.g., frequency, duration, intensity, level of severity) of the exercises were adapted to the patient’s abilities throughout the programme to support progression and continuous improvements in symptoms during the 12 weeks. The progression is guided using established tools such as the Borg scale of perceived exertion for aerobic exercise and the American College of Sports Medicine guidelines for strengthening exercises (e.g., the number of repetitions was added before increasing the number of sessions). During the exercise therapy programme, patients and physiotherapists discussed subjects related to self-management and individual participant goals, including a session supporting long-term self-management and behaviour change.
**9. Tailoring**	The programme is tailored to the characteristics, progression and improvements of the individual patient. Furthermore, the patients supported by the physiotherapists involved in the programme set individual goals that are followed up during the 12-week program.
**10. Modifications**	Not applicable, however, we will record whether modifications are needed during the testing of the intervention in the RCT as well as the type of modifications.
**11. How well (planned)**	The facilitators of the programme attended a specific one-day course to be certified to deliver the treatment programme to ensure programme fidelity. While there is no clear recommendation on how to train the facilitators, the physios delivering MOBILIZE are expert in delivering exercise therapy and self-management, therefore one day course was deemed appropriate and sufficient to deliver the intervention as intended. The programme focused on how to deliver and supervise the exercise therapy and self-management programme and support self-management and behaviour change in the individual. Adherence to the exercise and self-management sessions is measured by dividing the number of sessions attended by the total number of sessions offered. Monthly online fidelity meetings and in-person visits to the sites delivering the interventions are scheduled to assess fidelity. The focus of the fidelity meetings is to meet with the facilitators delivering the intervention to discuss the programme, including how it is being delivered at the sites and the success and the barriers/challenges that have been experienced
**12. How well (actual)**	Not applicable, however, we will record this as described in point 11.

*Described according to the Template for Intervention Description and Replication (TIDieR) and the Consensus on Exercise Reporting Template (CERT) items [67]

### Safety concerns

Behavioural interventions, including exercise therapy, are safe [68]. Although, in general, the risk of non-serious adverse events (according to the FDA definition [69], including short-lasting muscle pain and fatigue) may increase (19%), they do not increase the risk of serious adverse events such as hospitalisation and death [68]. Indeed, in people with multimorbidity they seem to reduce the risk of any serious adverse events by 38% [37]. However, the certainty of these findings in people with multimorbidity is low, mainly due to too few studies investigating this and the heterogeneous reporting of adverse events. Therefore, when developing the intervention, we tried to identify the possible adverse events associated with exercise therapy by looking at systematic reviews of exercise therapy for single chronic conditions [15, 18–20, 22, 29, 49, 68], national clinical guidelines [70], and by regular meetings with medical doctors specialised in the single chronic conditions. Overall, in people with one or more stable medical chronic conditions, it is contraindicated to exercise if they experience chest pain, uncontrolled hypertension or diabetes, irregular heartbeat, dizziness or sudden vision change [49]. We have instructed the facilitators delivering the intervention to check these contraindications before initiating any new exercise therapy session.

### The prototype of the intervention and amendments

Based on the evidence gathered and feedback from relevant stakeholders (listed in the patient and public involvement paragraph), we developed an intervention consisting of 18 (60 minutes) sessions of personalised, group-based supervised exercise therapy, six sessions of 90 minutes of group-based self-management support and six sessions of home-based unsupervised exercise therapy. The programme was designed to be delivered twice a week for 12 weeks and was tested in a mixed-methods feasibility study [34]. Briefly, the feasibility study showed the intervention was feasible and acceptable in people with multimorbidity in terms of recruitment rate, retention of outcomes at follow-up, outcome burden, adverse events, and qualitative feedback. Adherence to the home-based unsupervised exercise therapy, however, was low and a few barriers were identified in the interviews suggesting that some amendments were needed before proceeding to the RCT. For example, home-based exercise sessions were deemed challenging to complete by the patients, given the lack of supervision. Additionally, we discussed the option of splitting the self-management sessions into shorter but more frequent sessions to facilitate the uptake of the delivered information. Therefore, together with the physiotherapists, patient partners, project managers, and researchers of the MOBILIZE team, we amended the intervention by increasing the number of the group-based supervised exercise therapy sessions to 24 and the number of the group-based self-management sessions to 24.

Overall, each session of the intervention, which will be tested in a randomised controlled trial, lasts 90 minutes and includes 30 minutes of self-management support followed by 60 minutes of supervised group-based exercise therapy delivered by a physiotherapist. The material will be made publicly available in the randomised controlled trial paper. The overall content of the intervention is presented in Table 4 following the Template for the TIDieR (Template for Intervention Description and Replication) [71], incorporating the CERT (Consensus on Exercise Reporting Template) items [67], and S2 Table, including the mechano-biological descriptors of strengthening exercise therapy proposed by Toigo & Boutellier [72].

## Discussion

This paper summarises the development of the MOBILIZE intervention, which includes personalised exercise therapy and self-management support for people with multimorbidity. We followed the newest version Medical Research Council framework to develop the intervention [31], which targets people with multimorbidity and can be delivered in different settings at a relatively low cost as it uses material available in most gyms of hospitals, municipalities, and private physiotherapy clinics.

The evidence-based approach used to develop the MOBILIZE intervention together with the input from relevant stakeholders, resulted in an intervention for people with multimorbidity which includes components aimed to elicit physiological, psychosocial, and behavioural effects, considering contextual factors ultimately leading to improved health. This is novel for people with multimorbidity who are usually treated pharmacologically [26].

The iterative process used to develop and feasibility test the intervention helped us identify several challenges that could be addressed before the randomised controlled trial tests the effectiveness of the intervention. This is a key step in reducing waste in research [73].

The continuous interaction with key stakeholders to develop the MOBILZIE intervention in addition to improving the design of the intervention, aims to facilitate its implementation in clinical practice. While this approach has been acknowledged to be time-consuming, it is required when developing complex interventions aiming at improving people’s health behaviours [74].

### Limitations

The limited quality of evidence (few studies with inconsistent results) supporting the use of exercise therapy and self-management in people with multimorbidity meant that many decisions taken regarding the type, duration, and intensity of the intervention were taken from evidence of research for people with single chronic conditions. However, the extensive qualitative work conducted as part of the development of the intervention and the constant feedback from the stakeholders helped to adapt this knowledge to people with multimorbidity. This will also improve the translation of our research into clinical practice if the MOBILIZE intervention is shown to be superior to usual care in the randomised controlled trial.

## Conclusions

In this paper, we detail the development of a novel intervention for people with multimorbidity. The MOBILIZE intervention, is currently being tested for its safety and effectiveness in a randomised controlled trial. The intervention includes personalised exercise therapy and self-management support to improve health-related quality of life and physical function by eliciting physiological, psychosocial, contextual, and behavioural factors. The development of the MOBILIZE intervention has highlighted the importance of using an iterative process supported by the involvement of several stakeholders to identify solutions to the challenges of developing complex interventions.

## Supplementary Information

**Additional file 1.**

**Additional file 2.**

## Data Availability

The data reported in this paper are available on the Open Science Framework page of the MOBILIZE project https://osf.io/qk6yg/.

## Abbrevations

GUIDED: GUIDance for the rEporting of intervention Development
MRC: Medical Research Council Framework
BCt: Behaviour Change Techniques
OSF: Open Science Framework
