# Supplementary material for: Personalised exercise therapy and self-management support for people with multimorbidity: Development of the MOBILIZE intervention
Source: Pilot Feasibility Stud. 2022 Dec 2;8:244. doi: 10.1186/s40814-022-01204-y (PMC9717541; doi:10.1186/s40814-022-01204-y)
Supplement: Supplementary file 2 — Additional file 2. [file 40814_2022_1204_MOESM2_ESM.docx]

**S2 Table.** **Toigo & Boutellier checklis.** **Mechano-biological descriptors of strengthening exercise therapy stimuli in the MOBILIZE intervention.**

| **Brief name** | **Mechano-biological descriptors of strengthening exercise therapy stimuli.** |
| --- | --- |
| 1. Load magnitude | 5 to 7 on the OMNI scale. This perceived exertion scale includes numerical  categories from 0 (extremely easy) to 10 (extremely hard) |
| 2. Number of repetitions | 10 to 15 |
| 3. Number of sets | 2 to 3 |
| 4. Rest between sets | Thirty seconds, or until sufficiently recovered from previous set |
| 5. Number of exercise interventions | 24 sessions twice weekly |
| 6. Duration of the experimental period | 12 weeks |
| 7. Fractional and temporal distribution of the contraction modes per repetition and duration (s) of 1 repetition | Concentric phase (3 seconds) Eccentric phase (3seconds) Total duration of 1 repetition (6 seconds) |
| 8. Rest between repetitions | 60 seconds or until sufficiently recovered from previous set |
| 9. Time-under-tension (TUT) | Repetition TUT (6 seconds) Set TUT with 10 repetitions (60 seconds) and with 15 repetitions (90 seconds) Session TUT (2 sets × 10 repetitions=120 seconds); (3 sets × 12 repetitions=186 seconds) |
| 10. Volitional muscular failure | The participants are instructed to a ‘somewhat hard’ exertion. If volitional muscular failure occurs before the end of the set, the number of repetitions is adjusted so that the number of number of repetitions can be completed |
| 11. Range of motion | The patients are instructed to perform the exercises in full range of motion, if possible |
| 12. Recovery time between exercise sessions | The exercise sessions are held twice a week (e.g., Monday and Wednesday). So, there is one and four days of recovery in between the sessions |
| 13. Anatomical definition of the exercise (exercise form) | The participants are instructed to perform all the strengthening exercises with the perfect technique (full ROM and the same TUT, as described in items above). However, given the restricted mobility of most people with multiple chronic conditions (multimorbidity) this is not always possible. |

**S3 Table.** Contextual factors known to influence outcomes in clinical practice and how they were integrated in the MOBILIZE intervention.

| **Contextual factor in clinical practice^43^** | **How this is integrated in the MOBILIZE intervention** |
| --- | --- |
| Facilitator’s feature: professionalism, mindset, appearance. | The facilitators of the MOBILIZE intervention are physiotherapists with a Master’s degree in physiotherapy and clinical experience with people with chronic conditions. To deliver the MOBILIZE intervention they undergo a specific training as described in the TIDieR checklist. They are instructed to increase patients’ positive expectations such as adapt an authentic and empathic communication style, regularly assess and address patients’ anxieties, concerns, and treatment expectations, provide adequate information regarding disease, diagnoses, and treatments. While delivering the MOBILIZE intervention they wear a professional uniform. |
| Patient’s features: mindset, baseline level of symptoms, comorbidity, health condition, gender, age. | As part of the MOBILIZE intervention there is a 1 to 1, in person, initial session between the patient and the facilitator addressing patient’s expectations, goals, history and preferences to tailor the intervention. Additionally, the baseline physical characteristics of the participants are collected as described in the TIDieR checklist. |
| Patient-physiotherapist relationship: verbal and non-verbal communication. | The facilitators of the MOBILZIE intervention are instructed to adapt an authentic and empathic communication style when communicating with people with multimorbidity, including eye contact, and smiling. |
| Treatment features: therapeutic touch, modality, posology, marketing. | The MOBILIZE intervention is personalised, it is delivered by the same physiotherapist for the entire duration of the program and a campaign for recruitment has been launched in local and national newspapers with articles and video interviews. |
| Healthcare setting features: positive distractors, supportive indications, comfort element decorations and ornaments. | The MOBILIZE intervention is delivered in hospitals, municipalities, and private practices. All the settings use rooms with natural lighting, low noise level and an adequate temperature. Additionally, all the locations where the MOBILIZE intervention is delivered use supportive information regarding MOBILIZE (e.g., posters and flyers). |
